# Information and Communication Technologies to Support the Provision of Respite Care Services: Scoping Review

**DOI:** 10.2196/44750

**Published:** 2023-05-30

**Authors:** Aimee R Castro, Lydia Ould Brahim, Qirong Chen, Antonia Arnaert, Amélie Quesnel-Vallée, Karyn Moffatt, John Kildea, Vasiliki Bitzas, Carolyn Pang, Audrey-Jane Hall, Ariana Pagnotta, Argerie Tsimicalis

**Affiliations:** 1 Ingram School of Nursing McGill University Montreal, QC Canada; 2 School of Nursing Central South University Changsha China; 3 Department of Sociology McGill University Montreal, QC Canada; 4 Department of Epidemiology, Biostatistics and Occupational Health McGill University Montreal, QC Canada; 5 School of Information Studies McGill University Montreal, QC Canada; 6 Medical Physics Unit McGill University Montreal, QC Canada; 7 Geriatrics Unit, Palliative Care Unit CIUSSS West Central Montreal Montreal, QC Canada; 8 Oracle Corporation Redwood Shores, CA United States; 9 Société de Soins Palliatifs à Domicile du Grand Montréal Montreal, QC Canada; 10 Nursing Research Unit Shriners Hospitals for Children-Canada Montreal, QC Canada

**Keywords:** caregivers, eHealth, health services accessibility, home care services, mHealth, respite care, short break care

## Abstract

**Background:**

Respite care is one of the most frequently requested support services by family caregivers. Yet, too often, respite care services are inaccessible, due in part to families’ lack of knowledge regarding available services and a lack of service flexibility. Information and communication technologies (ICTs) may help to improve the flexibility of services available and families’ knowledge of such services. However, an understanding of the use of ICTs and research in this area is lacking.

**Objective:**

The objective of this study was to provide a comprehensive overview of the academic literature on ICTs for supporting the provision of respite care services.

**Methods:**

A scoping review study was conducted. Six library databases were systematically searched for relevant literature. Key data were extracted into a summary chart. Text and quantitative data were coded using descriptive qualitative content analysis techniques, and the results were collated and summarized into a comprehensive narrative.

**Results:**

A total of 23 papers describing 15 unique ICT programs exploring the potential of ICTs to support respite care services met the inclusion criteria. ICTs supported the provision of respite care by facilitating information-sharing with families and providers, recruiting and training respite care providers, and coordinating services. Key design considerations for developing respite care ICTs were trustworthiness and participatory design methods. Implementation considerations included designing for complementarity with existing services, assessing the appropriate timing for introducing the ICT-based services, and ensuring adequate promotion strategies to raise awareness about the services.

**Conclusions:**

There is limited but promising research on the potential of ICTs to support the provision of respite care services. Further research should be conducted to advance the results of this review, ultimately aiming to build ICTs that can improve the quality of, and access to, respite care services.

## Introduction

Respite care is one of the most frequently requested support services by family caregivers and is typically provided in person by a home care nurse or health care aide [1-3]. Respite care services are meant to help provide caregivers with short breaks from their caregiving responsibilities, so they may sustain their caregiving roles [2]. These breaks also offer patient and care receivers opportunities to socialize with new people and to access additional health care services through new care providers in their homes [2].

Unfortunately, respite care services are often underused, largely due to a lack of service flexibility and accessibility among respite care services capable of addressing different families’ unique needs [1,4,5]. Families may also lack information regarding the resources available to support them [6,7]. Family caregivers suggest that easier, more flexible access to respite care services would help support their caregiving work and alleviate feelings of burden [1,3].

Information and communication technologies (ICTs) have unique capabilities for supporting the flexible and efficient provision of community and home care services like respite care [8]. ICTs are tools that can be used to coordinate activities immediately over a distance and to facilitate the provision of flexible services [8]. Different forms of ICTs exist, such as personal computers, smartphones, and telephone systems [9]. The unique capabilities of ICTs could be used to make respite care services more flexible and accessible, by making it easier to coordinate care, share information about local respite care services, and provide continuing education to train more respite care providers [10-12].

A review of existing literature on technologies for supporting respite care services could be used to guide future research on developing ICTs to facilitate the provision of respite care services. Furthermore, a review can be particularly helpful for providing an overview of recommendations and trends from across multiple smaller research projects on ICTs for supporting respite care, when such recommendations and trends are not obvious in any single one of the smaller, context-specific studies. To our knowledge, no review has been conducted on respite care ICTs. Therefore, the aim of this scoping review was to provide a comprehensive overview of academic literature on ICTs for supporting the provision of respite care services.

## Methods

### Overview

A scoping review study was appropriate for our purposes, as this method allows researchers “to assess and understand the extent of the knowledge in an emerging field” (p. 2121) [13]. This study was conducted by adhering to the following key procedural steps for scoping review studies, as per the most recent JBI Manual for Evidence Synthesis [14].

### Defining and Aligning the Objective and Question

The primary research question was the following: what uses of ICTs have been studied in the academic literature for supporting the provision of in-person respite care services?

As per the JBI scoping review methodology, this question includes the following PCC (participant, context, concept) elements of a scoping review question: participant (stakeholders of respite care services, including family caregivers, patients, managers, and software designers), context (respite care services), and concept (ICTs) [14].

Related subquestions that we identified after iterative analyses were as follows: (1) what design factors should research teams consider when developing ICTs for respite care? (2) What implementation factors should research teams consider when developing ICTs for respite care, to support the uptake of ICTs?

### Developing and Aligning the Inclusion Criteria With the Question

The above research questions and PCC elements were used to identify our final inclusion criteria, as listed in Table 1.

**Table 1:** The inclusion and exclusion screening criteria.

Screening and inclusion criteria		Exclusion criteria
**Concept and phenomenon of interest**		
	ICTs^a^ that primarily support the provision of in-person respite care	Respite care that was not an in-person service (eg, if a robot or a video game were to be used to monitor or distract the care receiver temporarily) ICTs were primarily used for different purposes than respite care support (eg, telemedicine appointments with general practitioners, and general social media networks for caregivers to share their experiences)
**Participants and target end users of the ICTs**		
	All participants and target end users of the ICTs where care receivers had a medical or an aging concern	General parenting support services (eg, babysitting coordination) Respite as a service for people experiencing homelessness
**Context**		
	ICTs were being used to support the provision of respite care services located in the community (eg, in-home care, adult day-care centers, or short-term stays in long-term care institutions) Respite care had to be “in-person”; that is, another person would be physically present to provide care, allowing the caregiver to safely remove themselves from the care receiver’s environment	Remote presence (eg, in-home robotic tele-monitoring) Hospital-based care
**Research design**		
	All literature retrieved from academic library databases: empirical studies, editorials, commentaries, letters, abstracts, dissertations, perspectives, reviews, and study protocols	Full text was unavailable Study protocols without preliminary data were excluded
**Languages included**		
	English, French, and Chinese	Papers written in other languages would have been excluded However, no papers were excluded due to language because all retrieved sources were written in one of these three languages

^a^ICT: information and communication technology.

### Describing the Planned Approach

We did not submit a protocol for this scoping review for publication [14]. However, Multimedia Appendix 1 displays our Preferred Reporting Items for Systematic Reviews and Meta-Analyses (PRISMA) extension for Scoping Reviews checklist [15], summarizing our commitments to the scoping review methodology [13,14].

### Searching for the Evidence

A preliminary search strategy was conducted as follows: (1) reviewed by a research librarian [13,14]; (2) applied to MEDLINE (through OVID) and CINAHL Plus (through Ebscohost); and (3) refined and applied to MEDLINE and CINAHL, in addition to another 4 library databases: Embase Classic (1947-Present) (through OVID), APA PsycINFO (1987-Present) (through OVID), Scopus (through Elsevier), and Web of Science Core Collection (through Clarivate). A search strategy example for MEDLINE is outlined in Multimedia Appendix 2. The final comprehensive search was conducted in January 2022.

The reference lists of included publications and excluded ineligible literature reviews on respite care or technology for caregivers were also screened. Included publications were entered into Google Scholar to screen their “cited by” connections for inclusion. The Research Gate and Google Scholar profiles of the first and last authors were screened for references relevant to the research questions. The authors of respite care ICT programs established since 2010 were emailed seeking further information or updates on the projects that might not have been published yet [13].

### Selecting the Evidence

EndNote software was used to manage the search and remove duplicate entries [16]. Rayyan literature management software was used to independently screen all titles and abstracts, followed by reviewing the full texts of selected papers by 2 authors based on the inclusion criteria [14]. The screening process was iterative and at least 2 reviewers (AC and LOB or AP) discussed any challenges they encountered, refining the selection strategy as needed with input from coauthors.

### Data Extraction

One reviewer (AC) was responsible for charting data into an Excel (Microsoft Corp) workbook. Data items included year of publication, author, manuscript type, stated objectives, country, participant data, health condition necessitating caregiving, setting details, ICTs discussed (ie, intervention type), and other key results specific to our research questions [14]. Another reviewer (QC) independently extracted data from five manuscripts to verify the preliminary extraction process [14].

### Analysis of the Evidence

Descriptive qualitative content analysis techniques were used to code and relabel data into categories that addressed the research questions [13,14]. The full-text data from each manuscript (ie, introductions, methods, results, discussions, and any commentary data) were copied into Excel. Each row of data was open-coded to offer a brief summary of the main ideas for each data cell and to gain familiarity with the data. By rereading, comparing, and contrasting these open codes, we were able to generate a list of initial codes relevant to ICT uses, design, and implementation. The data were then relabeled in a new Excel column according to these initial codes. Using the “sort” function in Excel, similar ideas were grouped and regrouped in an iterative process as the codebook was refined to build new categories that we identified in the grouped data.

### Presentation of the Results and Summarizing the Evidence

We have presented the results using both, a table summarizing the extracted data and key results (Multimedia Appendix 3), and a text-based narrative of our results addressing the primary and secondary research questions in “a descriptive format that aligns with the objective/s and scope of the review” (p. 422) [14]. Multimedia Appendix 4 provides examples of raw data extracted from the publications that exemplify the key ideas addressing our research questions. 

## Results

### Search Results

**Figure 1:**
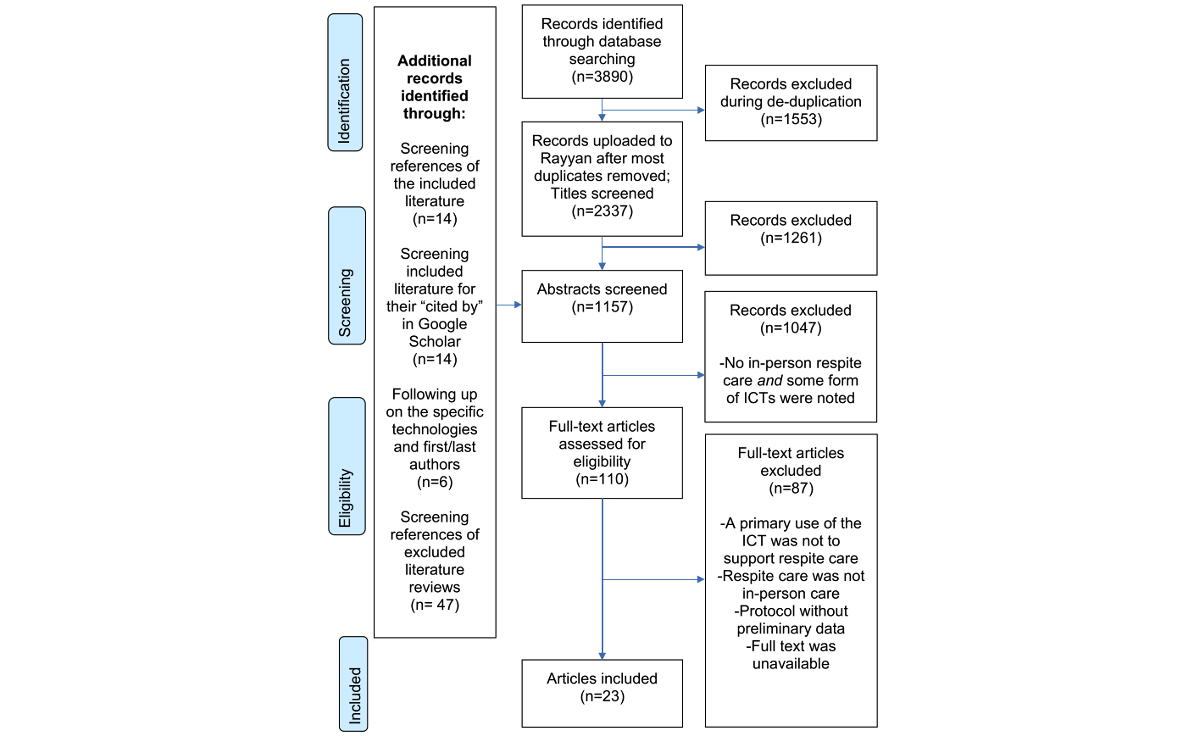
This flowchart outlines the results of the screening process. ICT: Information and communication technology.

Figure 1 shows the results of the screening process. Of the 3890 records screened, 23 met the inclusion criteria. All papers were published in English between 1990 and 2021.

### Study and Sample Characteristics

A total of 15 unique ICT research programs for exploring the uses of ICTs to support the provision of respite care services were described across the 23 papers (Multimedia Appendix 3). A total of 16 of the papers were empirical studies, including 6 qualitative [12,17-21], 6 quantitative [6,11,22-25], and 4 mixed methods [26-29] studies. Seven other papers provided project commentaries and overviews, or brief literature reviews [30-36].

Collectively, the 16 empirical studies included 2698 participants, although 74% (n=2000) of the total participants were derived from one survey study [23]. Participant types were typically family caregivers, health care providers, or health care stakeholders. Gender was only indicated in 6 studies, with women representing 76% (128/168) of these studies’ total participants. Age was indicated in 7 studies, with a range of 21-92 years of age, although most participants were middle-aged or older.

Additional details specific to program location, caregiving conditions necessitating respite care, and respite care settings discussed, are listed in Table 2.

**Table 2:** List of geographic locations, caregiving conditions, and respite care settings of the 15 programs.

Detailed criteria		Programs
**Geographic locations**		
	North America (United States only) programs (n=5)	Program 1 [33,34] Program 2 [11] Program 3 [24] Program 9 [35] Program 14 [19,21]
	Europe programs (n=7)	Program 4 [12,28] Program 5 [27] Program 6 [30] Program 7 [31,36] Program 10 [20] Program 11 [22,23] Program 15 [25]
	South America (Chile only) program (n=1)	Program 12 [17,18]
	Australia program (n=1)	Program 13 [6]
	Asia (Taiwan only) program (n=1)	Program 8 [26,29,32]
**Caregiving populations**		
	Older adults with chronic illnesses programs (n=8)	Program 1 [33,34] Program 3 [24] Program 4 [12,28] Program 7 [31,36] Program 12 [17,18] Program 13 [6] Program 14 [19,21] Program 15 [25]
	General chronic conditions, age unspecified program (n=1)	Program 11 [22,23]
	Children living with developmental disabilities programs (n=6)	Program 2 [11] Program 5 [27] Program 6 [30] Program 8 [26,29,32] Program 9 [35] Program 10 [20]
**Respite care setting**		
	In-home respite care programs (n=10)	Program 1 [33,34] Program 3 [24] Program 4 [12,28] Program 5 [27] Program 9 [35] Program 10 [20] Program 11 [22,23] Program 12 [17,18] Program 13 [6] Program 14 [19,21]
	Respite day care access programs (n=7)	Program 1 [33,34] Program 4 [12,28] Program 5 [27] Program 9 [35] Program 10 [20] Program 13 [6] Program 15 [25]
	Short-term stay planning programs (n=4)	Program 9 [35] Program 10 [20] Program 11 [22,23] Program 13 [6]

### Uses of Respite Care ICTs: Information-Sharing, Recruiting and Training, and Coordinating Care

ICTs were explored for the following uses in respite care services: (1) facilitating information-sharing with families and care providers, (2) offering recruitment and training resources for respite care providers, and (3) coordinating respite care services. Most programs focused on 1 of these 3 uses of ICTs for respite care, although one publication explored ICTs for both information-sharing and coordination [20]. Multimedia Appendix 3 describes key findings for these ICT uses in more detail.

### ICTs for Sharing Information About Respite Care Services

In 4 of the programs (5/23 publications), the use of text and video information about local respite care services was explored for supporting information-sharing with families and respite care providers [6,12,20,28,35]. ICTs for information-sharing included using televisions and remote controls to share modules about “Getting a Break” [12,28]; and using websites [6,20,35], social media [35], or telephone helplines [6] to learn more about local respite care services. However, 2 papers noted that for information on respite care services, families often relied on recommendations from their friends or clinicians, more than they relied on ICTs like the internet [6,20].

### ICTs for Recruiting and Training Respite Care Providers

Three programs (4/23 publications) used ICTs to support respite care provider recruitment and training [11,30,31,36]. One commentary briefly described the value of DVD materials for recruiting providers to serve ethnically diverse families [30]. Another research program studied the effectiveness of using a video-based training program to teach respite care skills to volunteers [11]. This program reported a mean improvement in the percentage of total correct responses on respite care skills questions after videotape training as between 35.5% and 47.6%, depending on the size of the training group [11]. Two publications described the creation of a CD and DVD training program to teach respite care students and staff how to provide high quality respite care services [31,36].

### ICTs for Coordinating Respite Care Services

Nine programs (15/23 publications) studied the uses of ICTs for facilitating respite coordination [17-27,29,32-34]. Authors noted that mobile devices with features, such as texting, location-based tracking, and mobile payment options, could improve the accessibility and flexibility of services by making communication and scheduling between families, respite care managers, and respite care providers easier [19-21,26,29,32,35].

Other programs explored the potential for ICTs to make service planning more efficient. For instance, information-sharing through interagency databases could improve service efficiency by ensuring that agencies have up-to-date information on service usage, to efficiently allocate their agencies’ resources [24,33,34]. Furthermore, several publications argued that ICTs, such as computerized databases, geographic information systems (GIS), and machine learning techniques, are often better at synthesizing large amounts of data than humans are. Authors suggested that these big data syntheses could be used for epidemiological studies and to predict and plan for community health initiatives, such as planning for different communities’ respite care needs [22,23,25,27,34].

### Design Considerations: Designing for Trust by Using Participatory Design Methods

Two key design considerations were noted across the research programs: the importance of designing for trust in the respite care services and their ICT platforms and the importance of using participatory design methods for developing these ICTs.

### Designing for Trust in the Providers, Services, and ICT Platforms

These ICT programs emphasized that for a respite care ICT to be useful, end users had to trust in (1) the providers delivering the respite care, (2) the service being facilitated by the platform, and (3) the ICT platform itself.

#### Trust in the Competencies of the Providers

End users had to believe that respite care providers being coordinated through a respite care ICT platform were competent and safe. Trust in the providers could be facilitated by sharing providers’ training experiences or institutional affiliations through the coordination platform [17,18].

The authors also emphasized that the ICTs should provide basic background information about the respite care service provider and the family receiving care, such as their care preferences and routines [17-19,21,26,29]. Easier communication with respite care agencies and providers using mobile devices and texting could further increase trust in the reliability and safety of respite care providers [17,19,21,26,29]. One program accomplished this through a quick check-in communication feature to facilitate communication between the family caregiver and respite care provider [19]. This program also recommended using elements of social matching (based on families’ and providers’ skills, preferences, and demographic features) to match the family with a suitable respite care provider [21]. Another program used the geo-location capabilities of ICTs to facilitate matching local and available respite care volunteers with families nearby in need of immediate respite care [32,37]. Other recommended features to engender trust in the programs’ respite care providers included background checks of respite care providers, training in community care ethics, and training in the fundamental skills of providing personal care [17,18,24,26,32].

#### Trust in the Reliability of the Service

End users needed to trust that suitable respite care services could be scheduled easily and reliably through the ICT platforms [6,17,19,31]. ICT features to support such scheduling included embedding the following features within the ICT platforms: scheduling assistants, respite care to-do lists (with task prioritization highlighted), lists of care receivers’ personal habits and family requirements for respite care services, and medical case files [19,26,29,32]. Features to enable calendar sharing, easy scheduling, and estimated arrival times also supported trust by helping to enhance the reliability of the service [19,32]. Additionally, a few programs recommended embedding a log feature to record respite care visit details and any additional notes or concerns for the family or future care providers to be aware of, to facilitate continuity of care [21,32].

#### Trust in the Data Privacy Standards and Usability of the Platform

Finally, end users had to trust in the data privacy standards and usability of the platform. End users needed to trust that their employees’ or families’ data recorded through the platform would remain protected and confidential [17,19,26,27,29,32,34]. Features to engender trust in ICT platforms included login modules that tracked where the sign-in occurred, and information exchange portals monitored by program administrators [21,26,29,32]. Two research programs suggested that for synthesizing large data sets of clients to plan services across respite care agencies, the patient or family data must first be deidentified [27,34]. End users also needed to trust that the platform would be useful and easy to use. For instance, 1 program added the option to leave voice recording notes as feedback, which was perceived as an easier input method than expecting users to type in notes [37]. To facilitate ease of use, the included programs particularly advocated the use of participatory design methods to build platforms that end users would trust.

### Using Participatory Design Methods to Build Usable and Trusted Platforms

The importance of designing ICTs with and for the end users (ie, family caregivers, patients or care receivers, or health care managers) was either stated explicitly in the papers or implicitly in the methods of including end users from the study onset [12,17,19,27,29,31-34,36]. A user-centered approach was explicitly used in 2 of the research programs [12,19,28]. Iterative testing with end users was implemented by at least 2 programs, to ensure that ICT platforms met end users’ needs [12,28,33,34].

A participatory approach was also evident in the designers’ consideration of the users’ comfort and ease with the technologies. Familiarity with the technology corresponded with end users’ willingness to use a new ICT for supporting respite care services [17,20,28]. One program suggested that older adults and family caregivers would be willing to use ICTs that they perceive to be helpful to them, such as television sets, remote controls, and telephone technologies to provide information and support on local respite care services [28]. Similarly, Foley [27] concluded that for GIS to be beneficial to health care planners, the planners must have a basic knowledge of GIS capabilities. The authors suggested that if ICTs are developed using tools that are less familiar to the end users, then developers should expect to spend additional time and resources to appropriately and efficiently train these service users [27,33,34].

However, while user-centered design and partnerships were emphasized, only 1 of the publications discussed participation by patients [28]; most of the 15 ICT programs focused on family caregivers, respite care providers, and respite care managers as the end users.

### Implementation Considerations for Respite Care ICTs

In addition to offering ICT design considerations, the 15 programs also offered considerations for successfully implementing the ICT platforms once they were developed. Specifically, the programs highlighted the importance of complementarity, timing, and promotion of respite care ICTs, to support ICT uptake.

### Considering Complementarity of the ICTs With Existing Services

Authors and participants noted that the ICTs being implemented should be designed to complement existing in-person respite care services, rather than to replace these services [6,11,12,17,20,21,27]. Several publications suggest that ICTs should facilitate, not replace, in-person contact with health care providers [6,17,20]. Similarly, Abarca et al [17] and Campos-Romero et al [18] noted that initial face-to-face meetings between volunteer respite care providers and families might be needed before these end users would be comfortable using the ICT to further coordinate respite care.

### Considering Timing and Family Readiness for Implementing the ICTs

Timing was an important factor in family caregivers’ willingness to use ICTs for respite care information and services [6,20,28]. McSwiggan et al [20] and Hanson et al [28] highlighted that the success of ICTs for accessing respite care depended on the caregiver’s stage of caregiving. For instance, at the early crisis stage of accepting the need for respite care, most caregivers relied on their social networks; they did not typically use ICTs or the Internet to find respite care information [20]. As they became more settled into their roles, caregivers also became more open to using ICTs [20]. Therefore, ICT developers must not only create the tool, but also assess when end users, such as respite care managers or families themselves, are most likely to be amenable to adopting the ICTs into their routines.

### Considering Promotion Strategies to Raise Awareness for Respite Care and the ICTs

Efficient promotion of novel services was also essential for addressing families’ needs for respite care and diminishing the burden of navigating ICT-based services [6,20]. The authors shared that when accessing respite care, caregivers often felt guilty or conflicted about needing these services, causing them to delay their search until a crisis occurred [6,20,21,28,31,36]. Once caregivers finally sought respite care, some found that adequate, flexible assistance was often difficult to find or unavailable [20,31]. Phillipson et al [6] concluded that launching a new ICT service was insufficient for supporting family caregivers and care receivers; frequent promotional strategies by the respite care services and primary health care providers are necessary when new ICTs are developed, to raise awareness of these respite care ICTs among families and care providers [6]. Such promotional strategies should include developers sharing the ICT links or platforms with families and health care providers; clinicians reminding families at regular primary care checkups that respite care services are available in their region; and clinicians reminding families that respite care services can improve both caregiver and patient well-being [6,20]. These strategies for promoting novel respite care services should be implemented as early in the caregiving journey as possible, so that families are made aware of resources before a crisis occurs [6,21].

## Discussion

### Overview

This scoping review analyzed 23 papers exploring how ICTs can support the provision of respite care services, providing a foundational map of the literature on respite care ICTs. The following discussion will compare our results to findings in related literature on ICTs for supporting other community health services. We will also discuss implications for future health care strategies and research on respite care ICTs.

### ICT Uses in Related Caregiving Services

Our scoping review found that ICTs can be used to support information-sharing about local respite care services with families and care providers, helping to raise awareness of existing services. Similarly, a cross-sectional questionnaire study of ICT-mediated support for family caregivers in the paid workforce found that 76.8% (86/129) of caregivers reported that access to information through the Internet about family caregiving support services was very valuable to them [38]. Another scoping review on ICT and non-ICT support for employed family caregivers also found that ICTs can be used to support information-sharing on caregiver supports like respite [39]. Therefore, our results add to the growing body of knowledge that ICTs may be particularly beneficial for supporting family caregivers by making information on respite care services more accessible.

ICTs can also support the building of caregiving skills, by offering more flexible and remote training structures than in-person training allows for. For example, ICTs, such as e-learning platforms and SMS text messaging have been found to be useful modalities for offering health care provider training in palliative care skills and supporting knowledge retention [40]. Thus, our results show that ICTs can be used for respite care skills training aligned with previous work in this area.

Finally, our review found that a common use of ICTs was for facilitating respite care coordination. Other researchers have also argued that ICTs can be used to support family caregiving by facilitating the coordination of caregiving support services [39,41]. Coordination support for home care nursing include easy SMS text messaging or calling members of the care team, as well as storing information on the care receiver’s health care status and caregiving support needs. This information could then be accessed digitally by new health care providers using secure ICT platforms [39]. Spann et al [39] did not mention the coordination of respite care directly in this context, but their results are likely transferred to the coordination of respite care services, which are a specific type of home care service. Furthermore, Andersson et al’s [38] study of ICT- and non–ICT-mediated caregiver supports found that family caregivers valued having assistance with planning and care coordination; yet, 79.4% of respondents did not receive such support from their care teams. Combined, our review and these other studies highlight the potential benefits of using ICTs to share information, provide training, and coordinate services to better support family caregiving.

### Design and Implementation Considerations for Related ICTs and Services

Design considerations for respite care ICTs identified in this study emphasized the need for trust, as well as the need for participatory design methods. Without trust in the respite care services, providers, and ICT platforms, family caregivers will not use the available resources [4,41,42]. In a recent scoping review on the challenges of using ICTs to support family caregiving, Hassan [43] concluded that facilitating trust in ICT was an important factor for successful ICT deployment. Trust in an ICT platform could be facilitated in a variety of ways, such as by working with end users and medical experts to co-design the ICTs, by teaching these end users how to assess the quality of health care ICTs, and by integrating the ICTs with complementary nondigital interactions (eg, face-to-face meetings) [43]. Furthermore, without participatory design methods, ICTs may be designed that do not actually meet the needs of family caregivers, health care workers, and care receivers; or that are not easy and efficient for these end users to use [8,43]. Thus, the conclusions of our scoping review on the importance of designing for trust with end users, and of using participatory design methods when designing respite care ICTs, are corroborated by external literature on ICTs for supporting caregiving.

Our review also found that if ICT developers did not plan for successful implementation within the existing health care context, well-designed ICTs might also not be taken up. Authors warned that ICT implementation was likely to fail for three reasons: (1) the ICT did not complement existing services, (2) it was not introduced to families at the appropriate times, and (3) it was inadequately promoted to existing services and families. Three other reviews on ICTs to support family caregiving also concluded that ICTs should complement, not replace, face-to-face services because families often feel that they uniquely benefit from face-to-face interactions with caregiving peers and health care teams [8,39,43]. Furthermore, respite care support, including ICT-based respite care tools, must be frequently promoted to family caregivers for early uptake, so that families have respite care resources in place before caregiving crises [1,39]. For ICTs to support these health care services, they must also be implemented with strategies to raise awareness of these programs among clinicians, families, and other stakeholders [43].

These design and implementation findings also speak to the importance of clinical-academic partnerships in ICT development for respite care [44]. Clinicians know that family caregivers and patients need more flexible and efficient respite care services [1,3]. Nurse clinicians can inform the design of complementary and useful ICT supports, which these clinicians can then promote with families and colleagues in their practices [43,44]. Furthermore, clinicians are best placed to assess timing and promotion of services that might help families. Clinicians should regularly update their knowledge of existing respite ICTs for families, frequently assess families’ readiness for such services, and regularly promote these services [2,39].

### Future Research Opportunities for Respite Care ICTs

There is limited but promising research on ICTs in respite care, as evidenced by the inclusion of only 23 papers despite our expansive search. Several of the papers touched on the same ICT respite care programs as other papers, with only 15 unique programs discussed. The studies often had small sample sizes and no control groups, as they were focused on ICT design and brainstorming with participants, rather than on conducting rigorous evaluations of the effects of ICT programs on respite care service outcomes. Such outcomes could include effects on caregiver and patient quality of life, service efficiency, or cost-effectiveness. Future research should not only describe the potential of ICTs to support respite care services but also evaluate the effectiveness of these programs in doing so.

Furthermore, ICTs have the potential to synthesize massive amounts of data. Yet, little work has been done to date to explore the potential of computerized data science tools (eg, GIS, machine learning) to facilitate the accessibility and delivery of respite care services using large health care data sets. Other technology evidence gaps in the academic literature include limited discussions of the potential of social media to support respite care information-sharing, training, and coordination; and little discussion of the use of ICTs for remote notification reminders of existing services. Given the importance that family caregivers placed on learning about respite care services from their peers and clinicians identified in our review [6,20], social media platforms may be important sources of peer-to-peer learning about caregiving support services [40]. Additionally, there was no discussion of ICT use for reminding families of available respite care services, such as using app notifications to remind families about the importance of beginning respite care services early in the caregiving role or to notify families of new respite services in their regions. Future research should build on these works to rigorously design and test the feasibility of smartphone apps for improving direct respite care coordination.

Finally, the participant demographics were relatively homogenous—the average age of included participants was often over 50 years and mostly focused on ICT support services to caregivers of aging adults. ICTs should be explored for their potential to support other specialized forms of respite care services, such as supporting families coping with cancer diagnoses, or families of younger adults with severe mental health challenges. Future research should consider the different perspectives of younger caregivers and care receivers and who will be using ICT-facilitated caregiving support services for many years to come [18,45,46]. Furthermore, only 1 program discussed care receivers as the participants or end users. For respite care ICT research to be truly user-centered in the designs and implementations, the perspectives of patients and care receivers should be included, as well [41,43,47,48].

### Strengths and Limitations

This review adhered to the most recent JBI scoping review methodology [14], and it was conducted across 6 library databases, allowing for a broad search and inclusion of relevant papers. Due to time and resource constraints, and several iterations of the protocol, we did not submit a protocol for this scoping review for publication [14]. However, we did submit a PRISMA checklist to support the rigor of our methods (Multimedia Appendix 1).

The original search was conducted in October 2019, and fully updated across the 6 databases in January 2022, making the comprehensive search for this review just over 1 year. The January 2022 search only returned 1 new manuscript. In February 2023, an abbreviated search was conducted across MEDLINE (through OVID), and limited to publications since January 1, 2022, using the following subject headings and search terms: (exp respite care/OR respite.tw,kf) AND (exp technology/OR (info* and communication* technolog*).tw,kf OR digital health.tw,kf). In MEDLINE, this search retrieved 4 references, none of which met the inclusion criteria. We conducted a similar search across CINAHL, and none of the retrieved references were eligible. Given these results and the limited resources of our team, we decided it would not be beneficial to reupdate the entire search.

The focus of this review on the academic literature means that the results of the included papers are evidence-based, reducing some of the risks of translating the conclusions of this review to clinical settings. However, by only searching academic databases, we may have missed uncatalogued but relevant gray literature (such as policy documents, or existing respite care smartphone apps). A forthcoming app store search study by our research team will help to address the latter limitation [49]. Finally, by keeping the search focused on the concept of “respite,” we may have missed literature that included respite but that was categorized under broader concepts, such as “palliative care” or “home care.” However, other systematic reviews on ICTs for palliative care [40,50] and ICTs for home care [8,51] have previously been conducted, offering complementary knowledge syntheses to this scoping review.

### Conclusions

This scoping review study adds to the bodies of academic literature on respite care services and ICTs by being the first study to offer an overview of the intersection of these 2 areas. This review establishes that there is limited but promising research on the potential uses of ICTs to support the provision of in-person respite care, by facilitating information-sharing, coordination, and training. However, for such ICTs to be successfully launched, they must be co-designed to engender trust, and they should be implemented with consideration for contextual concerns like complementarity, timing, and promotion. Additional research should be conducted to advance these conclusions and build ICTs for services that are designed with and for families needing respite care services, alongside the respite care organizations that serve these families. Patients and family caregivers want more flexible, trusted, and efficient respite care services; further research in this area should develop respite care ICTs to fulfill these needs.

## Appendix

Multimedia Appendix 1 PRISMA-ScR checklist for scoping reviews.

Multimedia Appendix 2 Search strategy for library database, MEDLINE.

Multimedia Appendix 3 Summary of 15 programs described in the 23 publications.

Multimedia Appendix 4 Examples of raw data coded into categories addressing the research questions.

## Abbreviations

GIS: geographic information system
ICT: information and communication technology
PCC: participant, context, concept
PRISMA: Preferred Reporting Items for Systematic Reviews and Meta-Analyses
