# Supplementary material for: Information and Communication Technologies to Support the Provision of Respite Care Services: Scoping Review
Source: JMIR Nurs. 2023 May 30;6:e44750. doi: 10.2196/44750 (PMC10265430; doi:10.2196/44750)
Supplement: Multimedia Appendix 2 [file nursing_v6i1e44750_app2.docx]

## Appendix 2: The search strategy for one library database, MEDLINE

| **Search number and topic** | **Search terms^a^** |
| --- | --- |
| **Search 1:**  **Subject headings (/), title words (.tw), and keywords (.kf) related to ICTs** | **Subject headings:**  exp computers, handheld/ OR exp mobile applications/ OR exp telemedicine/ OR exp cell phone/ OR exp smartphone/ OR exp computer systems/ OR exp information technology/ OR exp internet/ OR exp telephone/ OR exp technology/ OR exp information system/ OR exp text messaging/ OR exp computers/ OR exp telecommunications/ OR exp information science/ OR exp robotics/ OR exp social media/ OR exp virtual reality/  **Title words and keywords:**  OR smartphone*.tw,kf OR smart-phone*.tw,kf OR smart phone*.tw,kf OR mobile-app*.tw,kf OR mobile app*.tw,kf OR mhealth.tw,kf OR m-health.tw,kf OR telemedicine.tw,kf OR tele-medicine.tw,kf OR telehealth.tw,kf OR tele-health.tw,kf OR cellphone*.tw,kf OR cell-phone*.tw,kf OR cell* phone*.tw,kf OR mobile phone*.tw,kf OR ehealth.tw,kf OR e-health.tw,kf OR text* messag*.tw,kf OR mobile health.tw,kf OR apps.tw,kf OR handheld computer*.tw,kf OR hand-held computer*.tw,kf OR hand held computer*.tw,kf OR short messag* service*.tw,kf OR sms.tw,kf OR personal digital assistant*.tw,kf OR electronic health service*.tw,kf OR mobile devic*.tw,kf OR smart devic*.tw,kf OR texting.tw,kf OR info* technolog*.tw,kf OR communication* technolog*.tw,kf OR "info* communication* technolog*".tw,kf OR "info* and communication* technolog*".tw,kf OR ICTs.tw,kf OR internet.tw,kf OR telephone*.tw,kf OR tele-phone*.tw,kf OR phone*.tw,kf OR technolog*.tw,kf OR info* system*.tw,kf OR ((mobile or cell or smart) adj1 (phone* or app* or health or device*)).tw,kf OR ((info* or communication) adj1 (technolog* or system*)).tw,kf OR computer*.tw,kf OR digital tech*.tw,kf OR tele-communicat*.tw,kf OR telecommunicat*.tw,kf OR informatics.tw,kf OR wearable*.tw,kf OR smart-watch*.tw,kf OR smartwatch*.tw,kf OR smart watch*.tw,kf OR smart cloth*.tw,kf OR smart-cloth*.tw,kf OR robot*.tw,kf OR tele-rehab*.tw,kf or telerehab*.tw,kf OR remote consult*.tw,kf OR social media.tw,kf OR social network*.tw,kf OR website*.tw,kf OR virtual realit*.tw,kf |
| **Search 2:**  **Subject headings (/), title words (.tw), and keywords (.kf) related to respite care** | **Subject headings:**  exp respite care/  **Title words and keywords:**  OR respite.tw,kf OR (respite adj2 care*).tw,kf OR (care* adj2 break*).tw,kf OR (care* adj2 relief).tw,kf OR (short-term adj1 care*).tw,kf OR (short term adj1 care*).tw,kf  OR sitting service*.tw,kf OR adult day-care*.tw,kf OR adult daycare*.tw,kf OR adult day care*.tw,kf OR ((respite or relief or short-term or break*) adj2 care*).tw,kf |
| **Search 3:**  **Combining Search 1 and Search 2 using the “AND” operator** | [Search 1] AND [Search 2] |

^a^ Note: “exp” meant the subject heading was exploded to include sub-subject headings. MEDLINE uses * to search for concatenations of a term. For instance, “mobile app*.tw,kf” will search for any concatenations of this phrase among title word and keyword terms, such as “mobile applications” and “mobile apps.” MEDLINE “adjN” is used to search for words that are adjacent to each other by N terms.
