# Supplementary material for: Information and Communication Technologies to Support the Provision of Respite Care Services: Scoping Review
Source: JMIR Nurs. 2023 May 30;6:e44750. doi: 10.2196/44750 (PMC10265430; doi:10.2196/44750)
Supplement: Multimedia Appendix 3 [file nursing_v6i1e44750_app3.docx]

## Appendix 3: Summary chart of the 15 programs described in the 23 publications

| **Program & publications** | **Manuscript type** | **Sample size** | **Sample and/or setting details** | **Key findings** |
| --- | --- | --- | --- | --- |
| **Program 1: The Shared Information System Technology to Evaluate Respite Services (SISTERS) inter-agency database**  **Country:** United States  **ICTs discussed:** Personal computers, diskettes, SPSS data-entry and statistical software  **Health condition necessitating caregiving:** Alzheimer's disease or other forms of irreversible dementia  **Target end-users of the technology:**  Respite care agencies  **Support type:** *Coordination* via a personal-computer database to gather standardized information on the day-to-day operations of 6 local respite care agencies. | | | | |
| 1) ([Looman et al., 1990](#_ENREF_14)) | Commentary – Description of the process for developing the inter-agency database, with a short discussion of preliminary program results | Not applicable | 6 respite care agencies in Cuyahoga County, Ohio | The database revealed that 229 families across the agencies were accessing respite care services, that 45% of families used the day care service and 35% used in-home respite care, and that families coping with Alzheimer’s disease had higher support needs than other families.  The ongoing data syntheses from this inter-agency program were used to plan for future client needs, referral patterns, and supplementary services across the 6 agencies. |
| 2) ([Looman & Deimling, 1993](#_ENREF_13)) | Commentary - Project overview | Not applicable | 7 non-profit respite care agencies in Cuyahoga County, Ohio | The benefits of improved coordination to track clients and monitor service use across the agencies outweighed the challenges, such as time costs of data entry, and training employees on the database system.  The project was voluntarily continued by 4 of the 7 agencies beyond the project’s two-year funding deadline. |
| **Program 2: Videotape for training respite care service providers**  **Country:** United States  **ICTs discussed:** Videotapes  **Health condition necessitating caregiving:** Children living with developmental disabilities  **Target end-users of the technology:**  Respite care service agencies  Respite care service provider trainees  **Support type:** *Training* of respite care providers via a video-based instructional package. | | | | |
| 3) ([Neef et al., 1991](#_ENREF_17)) | Empirical – Quantitative studies | n=24 respite care service provider trainees | **Study 1 (preliminary testing):**  9 women  3 men  Age range:  21-52 years old  **Study 2 (clinical replication of Study 1):**  10 women  2 men  Age range: 19-58 years old | These studies reported improvement compared to baseline assessment in respite care skills (such as managing children’s routines and communicating with parents) of trainees following the video-tape curriculum in different formats, compared to their baseline knowledge.  The mean increase in the percentage of total correct responses on skills questions after videotape training in the clinical replication experiment was 35.5% for the individual format group, 47.6% for the partner format group, and 43.6% for the group format trainees. |
| **Program 3: The older volunteer service bank**  **Country:** United States  **ICTs discussed:** Personal computers, a service credit software platform  **Health condition necessitating caregiving:** Frail older adults living in the community  **Target end-users of the technology:**  Volunteers providing respite care services to families of older adults  **Support type:** *Coordination* via a computerized service credit system that tracked volunteer respite care service providers’ hours of care, which volunteers could then redeem to receive respite care services for their families in the future. | | | | |
| 4) ([Ozawa & Morrowhowell, 1993](#_ENREF_18)) | Empirical – Quantitative study | n=263 volunteers | 37 respite care volunteer agencies  Average age: 71 years old | Volunteers reported high satisfaction with the program.  The mean number of volunteer respite hours tracked since program inception (i.e., 3 years prior) was 52 hours. |
| **Program 4: The Assisting Carers using Telematic Interventions to meet Older person’s Needs (ACTION) project**  **Country:** Pan-European program: Sweden, Portugal, England, Northern Ireland, and the Republic of Ireland  **ICTs discussed:** Personal computers, software, televisions, and video-telephony equipment  **Health condition necessitating caregiving:** Frail older adults living at home  **Target end-users of the technology:**  Community health care providers Family caregivers of older adults  Older adults  **Support type:** *Information sharing* via multimedia programs developed through the ACTION project for educating families, by providing information and support on available respite care services, financial supports; and by providing links in the videophone program for family caregivers to talk with professional caregivers and other family caregivers taking part in the project. | | | | |
| 5) ([Hanson et al., 1999](#_ENREF_11)) | Empirical –  Qualitative methods | Total n=10 participants | **2 peer reviewers** independently assessed the programs’ designs (1 nurse expert in health information technology, 1 key service purchaser /provider)  **8 user trial participants for evaluating program materials:**  By role:  4 professional carers  4 family caregivers  By gender:  4 women  4 men  Age range:  30-85 years old | Peer reviewers gave overall positive reviews of the multimedia programs.  Preliminary participant feedback suggested the program could help to address the information and support needs of family caregivers and older adults regarding respite care and long-term care planning.  User trial participants supported the iterative refinement of the ACTION programs by compiling a problem list for corrections (e.g., enlarging the buttons for those with arthritis, providing more examples of financial benefits or costs of services, and using lay terminology). |
| 6) ([Hanson et al., 2000](#_ENREF_12)) | Empirical - Mixed methods | n=148 participants | 43 family caregivers of older adults  14 older adult care-receivers  91 professional care providers | 78-94% of the 148 respondents recorded high levels of satisfaction for the presentation, wording of the programmes, and ease of learning and navigation.  Qualitative feedback supported these positive quantitative evaluations.  Participants shared that they appreciated having information about local respite care services in one place, and that the programs were easy to navigate using the familiar technologies of televisions and remote controls. |
| **Program 5: Using Geographic Information Systems (GIS) for supporting short-term care service planning**    **Country:** England  **ICTs discussed:** Personal computers, GIS software  **Health condition necessitating caregiving:** Children living with severe disabilities  **Target end-users of the technology:**  Respite care service providers  Healthcare planners  **Support type:** *Coordination* via GIS software to gather information on which respite care services were being offered in different locations, and to compare service usage levels to families’ needs in those regions. | | | | |
| 7) ([Foley, 2002](#_ENREF_10)) | Empirical - Mixed methods | n=52 participants | **Individual semi-structured interview participants:**  n=27 individual semi-structured interviews:  -5 from the health sector  -9 from the social service sector  -6 from the special education sector  -5 from the voluntary sector  -1 from the private sector  -1 from an external GIS unit  **Focus group 1:**  15 participants from social services and a caregiving lobbying group  **Focus group 2:**  10 participants from primary care groups in Sussex | Participants perceived GIS to be a useful complementary tool for planning short-break care services, by helping to synthesize large amounts of respite care data to create geo-spatial maps for visualization and service planning.  Participants shared particular concerns regarding the quality of and access to data for informing GIS modelling. |
| **Program 6: "Welcome to the Family” DVD for recruiting culturally safe respite care providers**  **Country:** England  **ICTs discussed:** DVD  **Health condition necessitating caregiving:** Children living with disabilities  **Target end-users of the technology:**  Potential respite care providers  Families of children living with disabilities  **Support type:** *Training* via sharing videos of family-based stories to recruit short-break carers from diverse ethnic backgrounds for disabled children to help give families respite. | | | | |
| 8) ([Cole, 2008](#_ENREF_7)) | Commentary – Personal review of a respite care technology | Not applicable | Not applicable | The DVD was perceived to be valuable to families of children with disabilities and could help recruit more diverse respite care providers who could better meet families' diverse needs, by recruiting short-break carers who speak the same language or those who share the same beliefs and/or cultures. |
| **Program 7: A CD-ROM / DVD providing information and suggestions for respite care agency improvement and family access**  **Country:** England  **ICTs discussed:** CD-ROM, later DVD  **Health condition necessitating caregiving:** Older adults living with dementia  **Target end-users of the technology:**  Respite care agencies  Families of older adults with dementia  **Support type:** *Training* via the creation of a CD and DVD training program to teach respite care students and staff how to provide high quality respite care services | | | | |
| 9) ([Ryan et al., 2008](#_ENREF_22)) | Commentary – Project overview for the CD-ROM | Not applicable | Not applicable | The authors suggested that the CD-ROM would benefit future healthcare students and respite care providers in learning how to care for families coping with dementia. |
| 10) ([De Soysa et al., 2010](#_ENREF_9)) | Commentary – Project overview for the DVD | Not applicable | Not applicable | Developing the ICT was more difficult than the team expected, due to families needing coaching in how to share their stories on video camera, and due to time delays in filming and production.  However, the authors suggested that the resource would ultimately support the development of better respite care services in their communities, by training healthcare providers in how to provide high-quality respite care services to families coping with dementia. |
| **Program 8: A mobile platform for coordinating volunteer respite care services to families of children living with developmental disabilities**  **Country:** Taiwan  **ICTs discussed:** Mobile devices, personal computers, and personal digital assistants; coordinating platform software  **Health condition necessitating caregiving:** Children living with developmental disabilities  **Target end-users of the technology:**  Parents of children living with developmental disabilities Volunteers providing respite care services  Volunteer respite care service managers  **Support type:** *Coordination* via a mobile platform to identify and match local respite care volunteers to families’ needs, and to support communication between families and volunteers | | | | |
| 11) ([Chou et al., 2008](#_ENREF_5)) | Commentary - Project overview | Not applicable | Not applicable | With ongoing discussions with families and the engineering team, a mobile and web-based system was developed for coordinating respite care that families and agencies perceived to support their needs. |
| 12) ([Yang, 2009](#_ENREF_23)) | Empirical –  Mixed methods study | **Design interviews:**  n=25  family members of children living with developmental disabilities  **Satisfaction questionnaire:**  Numbers not reported | No further details provided | 196 respite care and support service matches were made using the platform between November 2008 and June 2009.  Based on questionnaires sent out to families between 2008-2009, family satisfaction with the service rose from 3.8/5 to 4.84/5 during that period. However, the number of questionnaires sent out and received were not reported.  Participants and the research team concluded that the final system was considered practical to implement and could improve the delivery of respite care services. |
| 13) ([Chou et al., 2011](#_ENREF_6)) | Empirical –  Mixed methods study | n=32  participants  **Design interviews:**  3 managers (2 executives and parents of a child living with developmental disabilities, 1 technical assistant)  **Evaluation questionnaires:**  15 parents of children living with developmental disabilities  14 volunteers (social workers, students, and respite care staff members) | No further details provided | Overall satisfaction with the program improved from an average score of 3.58/5 to 4.26/5 over 3 years of implementation.  The computerized system provided a practical tool for accessing timely respite care services.  The system also offered social networking opportunities and facilitated information exchange among participants on the platform. |
| **Program 9: A literature review on the uses of the Internet to support family caregivers, including for accessing respite care services**  **Country:** United States  **ICTs discussed:** Internet-connected personal computing devices, websites for local respite care services, online support groups, and approval ratings for respite care  **Health condition necessitating caregiving:** Frail older adults living in the community  **Target end-users of the technology:**  Family caregivers  **Support type:** *Information sharing* via Internet-based services for families to learn about local respite care services | | | | |
| 14) ([Petrovic, 2013](#_ENREF_19)) | Commentary – Short literature review | Not applicable (short commentary) |  | The author argued that the Internet offers important opportunities to better support family caregivers in need of respite care, by creating space for social media platforms to share respite care information, by hosting peer-support groups online, by facilitating videoconferencing group support interventions, and by hosting databases for listing local respite care services.  The Internet may be a particularly important resource for family caregivers who have difficulties leaving the home environment due to their care responsibilities. |
| **Program 10: Exploring information-sharing strategies in respite care services**  **Country:** Scotland  **ICTs discussed:** Personal computers, telephone systems, mobile phones, Internet-connected devices, online care plans  **Health condition necessitating caregiving:** Older adults living with cognitive impairments  **Target end-users of the technology:**  Family caregivers  **Support type:** *Information sharing & coordination* via the use of information and communication technologies, such as websites and mobile phones | | | | |
| 15) ([McSwiggan et al., 2017](#_ENREF_15)) | Empirical - Qualitative study | n=24 caregivers of older adults | Gender:  17 women 7 men | Participants shared that ICTs offered an important source of information about respite care services to family caregivers; but traditional non-ICT information sharing methods, such as through face-to-face meetings with primary care providers, were also needed to make respite care accessible to families.  Mobile phones were very important for families to coordinate with respite care services. |
| **Program 11: Computer simulations for predicting respite care costs and caregiver burnout**  **Country:** France  **ICTs discussed:** Personal computers, software for agent-based modeling and simulation using Markov modeling and machine learning techniques  **Health condition necessitating caregiving:** Patients with chronic diseases living in the community  **Target end-users of the technology:**  Respite care service planners  **Support type:** *Coordination* via the use of computer simulations to predict local caregiver burden and subsequent respite care demand | | | | |
| 16) ([Batata et al., 2017](#_ENREF_2)) | Empirical – Quantitative study | Fictitious dataset of family caregiver traits, generated based on a monthly data set of 400 caregivers over one year detailing the health status of the patient and the caregiver’s perceived burden and quality of life | Not stated | The training resulted in a computer model that was able to predict optimal capacity of respite care structures for the test set.  For quality of service, when the capacity of the local non-emergency respite care services increased, the model predicted that the monthly rate of emergency respite care use would decrease significantly as caregiver burden was reduced, lowering overall costs. |
| 17) ([Batata et al., 2018](#_ENREF_3)) | Empirical – Quantitative study | n=2,000 respondents from an online caregiving survey | No further sample data provided | The neural networks developed based on this dataset predicted caregiver burnout and optimal respite care admissions policies.  Computer models such as the one developed could be useful for predicting caregiver burnout and risk for expensive emergency respite care placements. |
| **Program 12: Exploring perspectives on the uses of ICTs to coordinate volunteer respite care services**  **Country:** Chile  **ICTs discussed:** Common feature phone and/or a device with an Internet connection  **Health condition necessitating caregiving:** Bedridden older adults  **Target end-users of the technology:**  Informal caregivers  Volunteer respite care service providers  **Support type:** *Coordination* via the potential capabilities of ICTs to match families with volunteer respite care providers, and to allow them to communicate with each other. | | | | |
| 18) ([Abarca et al., 2018](#_ENREF_1)) | Empirical – Qualitative study | n=10 family caregivers of bedridden older adults | Gender:  9 women 1 man  Average age:  53.7 years  (Range: 38–77 years old) | Family caregivers living in low-income neighborhoods believed that ICTs could be helpful for accessing respite care services by facilitating a peer-to-peer economy system, and by facilitating communication and sharing important background information on families and volunteers.  However, due to some participants’ lack of familiarity with smartphones, there was some disagreement as to whether the program should be delivered using a smartphone application, versus using a traditional offline feature phone. |
| 19) ([Campos-Romero et al., 2020](#_ENREF_4)) | Empirical – Qualitative study | n=8 volunteers for respite care services | Average age: 49.1 years  (Range: 23-65) | Participants believed that with trusted institutional backing and technologies that families were comfortable with, an ICT could be acceptable to volunteers for coordinating respite care with families in low-income neighborhoods.  Such an ICT could give multiple options for communication to establish respite care relationships and continuity of care, via phone calls, text messages, WhatsApp messages, emails, and/or written or voice recording notes. |
| **Program 13: Assessing family caregivers’ knowledge of respite care services, after the launch of national ICT-based programs to support family caregiving**  **Country:** Australia  **ICTs discussed:** Personal computers, telephones, Internet-connected devices    **Health condition necessitating caregiving:** Older adults living with various dementias  **Target end-users of the technology:**  Family caregivers  **Support type:** *Information sharing* via several ICT-based programs for sharing respite care services and helping families to connect with those services: My Aged Care (telephone line and website), Carelink and Carer Respite Services (helpline and website), and Carer Gateway (helpline and website) | | | | |
| 20) ([Phillipson et al., 2019](#_ENREF_20)) | Empirical – Quantitative study | n=84 family caregivers of people living with dementias | Gender:  62 women 22 men  Mean age:  70 years old  (Range: 38-92 years old) | 86% of participants had sought respite care information in the previous 12 months, with most seeking information from sources such as their primary clinicians or family, rather than from ICTs.  The My Aged Care website was used by 25% of participants to find information about respite care services. The help-line for short-term and emergency respite was used by 35% of participants.  The new government ICT initiatives were inadequately addressing family caregivers’ needs for information and support, largely due to families’ lack of awareness of such services and to the need for stronger community-based messaging from clinicians promoting the positive benefits of respite care services. |
| **Program 14: Designing a smartphone application (“app”) prototype to facilitate respite care coordination**  **Country:** United States  **ICTs discussed:** Smartphone application prototype design  **Health condition necessitating caregiving:** Aging-related and other degenerative health conditions  **Target end-users of the technology:**  Family caregivers  Respite care service providers  **Support type:** *Coordination* via a mobile application for supporting scheduling and communication of respite care services | | | | |
| 21) ([Currin et al., 2019](#_ENREF_8)) | Empirical - Qualitative user-centered design research | n=18 participants | **Participant roles:**  7 primary family caregivers of older adults with degenerative conditions 8 respite care providers 3 participants who were both caregivers and respite care providers)  **Gender:** 17 women 1 man  **Age range:**  19-80 years old | This user-centered design study resulted in the design of a smartphone application prototype to coordinate respite care services.  Future user-testing of the prototype was planned. |
| 22) ([Min et al., 2020](#_ENREF_16)) | Empirical - Qualitative design research | Same participants as for ([Currin et al., 2019](#_ENREF_8)) | Same participants as for ([Currin et al., 2019](#_ENREF_8)) | Design considerations for engendering trust in a respite care app included:  -Easy check-in with caregivers, to build trust in the new and temporary relationship between family caregiver and respite care provider  -Background information on the care-receiver  -Profile reviews of potential respite care providers by previous families/customers, for new families to screen  -Elements of social matching (skills, preferences, and demographic features) to match the family with a suitable respite care provider  Design considerations for supporting communication included:  -Communication channels  -Integration with other smartphone apps, smart home systems, and wearable technology (e.g. for health status updates)  -Log entries sharing basic information about the visit  -Lists of essential tasks for the respite care provider to complete, in order of priority |
| **Program 15: Using GIS mapping software to determine the geographic availability and accessibility of day care services for people with dementia in Ireland**  **Country:** Ireland  **ICTs discussed:**  GIS software: ESRI® ArcGIS® ArcMap™ version 10.2  Ungeneralised (high-resolution) administrative boundary shapefiles for Ireland from the Central Statistics Office  **Health condition necessitating caregiving:** Dementia  **Target end-users of the technology:**  Policy makers and healthcare administrators determining where to build future adult day care programs  **Support type:** *Coordination* via inputting large datasets such as national survey data into GIS software, to assess dementia day care accessibility (i.e., distance) and availability (i.e., number of spaces versus population density), in order to determine where to build future day care programs. | | | | |
| 23) ([Pierse et al., 2020](#_ENREF_21)) | Empirical -  Quantitative secondary analysis of national survey daycare centre data | Survey data were based on responses from 317 day care centres across Ireland | Not applicable | Results revealed that 18% of people living with dementia in Ireland did not live an accessible distance from their nearest day care centre.  The GIS methodology could allow policy makers to better interpret large datasets, combining accessibility and availability data for current services to predict where to best allocate future services based on need. |

## References

Abarca, E., Campos-Romero, S., Herskovic, V., & Fuentes, C. (2018). Perceptions on technology for volunteer respite care for bedridden elders in Chile. *Int J Qual Stud Health Well-being*, *13*(1), 1422663. <https://doi.org/10.1080/17482631.2017.1422663>

Batata, O., Augusto, V., Ebrahimi, S., & Xie, X. (2017). Performance evaluation of respite care services through multi-agent based simulation. *Proceedings of the 2017 Winter Simulation Conference*, 2904-2916. <https://doi.org/10.1109/WSC.2017.8248013>

Batata, O., Augusto, V., & Xie, X. (2018). Mixed machine learning and agent-based simulation for respite care evaluation. *2018 Winter Simulation Conference (WSC)*, 2668-2679. <https://doi.org/10.1109/WSC.2018.8632385>.

Campos-Romero, S., Herskovic, V., Fuentes, C., & Abarca, E. (2020). Perceptions on connecting respite care volunteers and caregivers. *Int J Environ Res Public Health*, *17*(8), 2911. <https://doi.org/10.3390/ijerph17082911>

Chou, L. D., Lai, N. H., Chen, Y. W., Chang, Y. J., Huang, L. F., Chiang, W. L., Chin, H. Y., & Yang, J. Y. (2008). Management of mobile social network services for families with developmental delay children. *2008 10th IEEE International Conference on E-Health Networking, Applications and Services*, 79-+. <https://doi.org/10.1109/HEALTH.2008.4600115>.

Chou, L. D., Lai, N. H., Chen, Y. W., Chang, Y. J., Yang, J. Y., Huang, L. F., Chiang, W. L., Chiu, H. Y., & Shin, H. Y. (2011). Mobile social network services for families with children with developmental disabilities. *IEEE Trans Inf Technol Biomed*, *15*(4), 585-593. <https://doi.org/10.1109/TITB.2011.2155663>

Cole, A. (2008). Review: Welcome to the family. *Learning Disability Today*, *8*(1), 49-49. <https://proxy.library.mcgill.ca/login?url=http://search.ebscohost.com/login.aspx?direct=true&db=rzh&AN=105703360&site=ehost-live>

Currin, F., Min, A., & Razo, G. (2019). Give me a break: Design for communication among family caregivers and respite caregivers. *Extended Abstracts of the 2019 Conference on Human Factors in Computing Systems (CHI)*, 1-6. <https://doi.org/10.1145/3290607.3309687>

De Soysa, R., Grayson, P., Grayson, J., Ryan, T., & Nolan, M. (2010). Telling our story: Good practice in respite care. *Journal of Dementia Care*, *18*(6), 12-13. <https://proxy.library.mcgill.ca/login?url=http://search.ebscohost.com/login.aspx?direct=true&db=rzh&AN=104965217&site=ehost-live>

Foley, R. (2002). Assessing the applicability of GIS in a health and social care setting: Planning services for informal carers in East Sussex, England. *Soc Sci Med*, *55*(1), 79-96. <https://doi.org/10.1016/s0277-9536(01)00208-8>

Hanson, E. J., Tetley, J., & Clarke, A. (1999). A multimedia intervention to support family caregivers. *Gerontologist*, *39*(6), 736-741. <https://doi.org/DOI> 10.1093/geront/39.6.736

Hanson, E. J., Tetley, J., & Shewan, J. (2000). Supporting family carers using interactive multimedia. *British journal of nursing (Mark Allen Publishing)*, *9*(11), 713-719. <https://doi.org/10.12968/bjon.2000.9.11.6262>

Looman, W., & Deimling, G. (1993). The maturation of a multiagency computerization effort for Alzheimer's respite services. *Computers in Human Services*, *9*(1), 97-110. <https://doi.org/10.1300/J407v09n01_13>

Looman, W., Noelker, L., & Deimling, G. (1990). Using information system technology to coordinate specialized services for the elderly. *Proceedings of the Conference on Computers and the Quality of Life* 106-111. <https://doi.org/10.1145/97344.97399>

McSwiggan, L. C., Marston, J., Campbell, M., Kelly, T. B., & Kroll, T. (2017). Information-sharing with respite care services for older adults: A qualitative exploration of carers' experiences. *Health Soc Care Community*, *25*(4), 1404-1415. <https://doi.org/10.1111/hsc.12440>

Min, A., Currin, F., Razo, G., Connelly, K., & Shih, P. C. (2020). Can I take a break? Facilitating in-home respite care for family caregivers of older adults. *American Medical Informatics Association Annual Symposium Proceedings (AMIA ’20)*. <https://www.ncbi.nlm.nih.gov/pmc/articles/PMC8075491/>

Neef, N. A., Trachtenberg, S., Loeb, J., & Sterner, K. (1991). Video-based training of respite care providers: An interactional analysis of presentation format. *J Appl Behav Anal*, *24*(3), 473-486. <https://doi.org/10.1901/jaba.1991.24-473>

Ozawa, M. N., & Morrowhowell, N. (1993). Missouri service credit system for respite care: An exploratory-study. *Journal of Gerontological Social Work*, *21*(1-2), 147-160. <https://doi.org/10.1300/J083V21N01_10>

Petrovic, K. (2013). Respite and the internet: Accessing care for older adults in the 21st century. *Computers in Human Behavior*, *29*(6), 2448-2452. <https://doi.org/10.1016/j.chb.2013.02.005>

Phillipson, L., Johnson, K., Cridland, E., Hall, D., Neville, C., Fielding, E., & Hasan, H. (2019). Survey of knowledge of respite services: Knowledge, help-seeking and efficacy to find respite services: An exploratory study in help-seeking carers of people with dementia in the context of aged care reforms. *BMC Geriatrics*, *19*(1), 2. <https://doi.org/https://doi.org/10.1186/s12877-018-1009-7>

Pierse, T., Keogh, F., O'Shea, E., & Cullinan, J. (2020). Geographic availability and accessibility of day care services for people with dementia in Ireland. *BMC Health Serv Res*, *20*(1), 476. <https://doi.org/10.1186/s12913-020-05341-z>

Ryan, T., Noble, R., Thorpe, P., & Nolan, M. (2008). Out and about: A valued community respite service. *Journal of Dementia Care*, *16*(2), 34-35. <https://proxy.library.mcgill.ca/login?url=http://search.ebscohost.com/login.aspx?direct=true&db=rzh&AN=105739823&site=ehost-live>

Yang, J.-Y. (2009). A respite care information system for families with developmental delay children through mobile networks. *Proceedings of the 11th international ACM SIGACCESS conference on Computers and accessibility*, 261-262. <https://doi.org/10.1145/1639642.1639706>
