# Supplementary material for: Information and Communication Technologies to Support the Provision of Respite Care Services: Scoping Review
Source: JMIR Nurs. 2023 May 30;6:e44750. doi: 10.2196/44750 (PMC10265430; doi:10.2196/44750)
Supplement: Multimedia Appendix 4 [file nursing_v6i1e44750_app4.docx]

## Appendix 4: Examples of raw data coded into categories addressing the research questions

| **Major and minor categories** | | **Example** |
| --- | --- | --- |
| **Uses of Respite Care ICTs: Information-sharing, Recruiting and Training, and Coordinating Care** | | |
| ICTs for sharing information about respite care services | “Use of information and communication technologies to promote information-sharing with respite services would benefit from further exploration.”   ([McSwiggan et al., 2017](#_ENREF_5)) (p. 1404) | |
| ICTs for recruiting and training respite care providers | “The savings in trainer time and cost that accrue through the use of packaged videotape instructional programs could be used to provide remedial training in individualized areas of need. Only 15 to 45 min of remedial training were required for any of the participants to reach criteria. (This does not include, however, the 30- to 60 min simulation probes used to assess performance.)”   ([Neef et al., 1991](#_ENREF_7)) (p. 484) | |
| ICTs for coordinating respite care services | “According to the results of this study, potential volunteers are willing to use ICT to establish communication with caregivers and to ensure respite is achieved.”   ([Campos-Romero et al., 2020](#_ENREF_1)) (p. 8) | |
| **Design Considerations: Designing for Trust by Using Participatory Design Methods** | | |
| Designing for trust in the providers, service, and ICT platforms | **Providers:**  “One [professional respite care provider] observed the importance of being able to communicate both face-to-face and electronically during the early phase of the relationship-building process. She has utilized a smartphone app to chat, send messages, and share memos with [primary family] caregivers.  *P12 (respite professional caregiver): ‘We want to make sure we have good relationships with families, and so it’s all about building trust. You don’t start off that way when people start coming into our program. It gets built over time. ... Sometimes people will tell you more in the chat or text message function rather than in person.’”*   ([Min et al., 2020](#_ENREF_6)) (p. 853)  **Service:**  “Having shared information with services about the type of respite care that would best suit their needs, carers were disappointed and frustrated when their expectations of accessible and equitable services could not be accommodated. Those living in rural locations, in particular, found that they had little choice in terms of location and types of respite care available to them.”   ([McSwiggan et al., 2017](#_ENREF_5))(p. 1407)    **ICT platform:**  “Since this system guarantees the security of location information and it also verifies user credentials, mobile users can ubiquitously access MSNSM system through Transmission Con­trol Protocol/Internet Protocol and Hypertext Transfer Protocol (HTTP).”   ([Chou et al., 2011](#_ENREF_2)) (p. 587) | |
| Using participatory design methods to build a usable and trusted platform | “Collaboration has once again been a central feature in the development of the CD-ROM, based on the good communication that exists between the three main groups involved. This was necessary to help plan and co-ordinate the complex arrangements required for filming, finalising student tasks and preparing families for the arrival of film and production teams. Preparatory work with people with dementia and families was essential for the implications of their involvement to be fully understood, including the fact that their experiences would help shape the content of the documentary material. Academic and practice partners worked together in establishing a consent process for people with dementia involved in the filming.”   ([Ryan et al., 2008](#_ENREF_9)) (p. 35) | |
| **Implementation Considerations for Respite Care ICTs** | | |
| Considering complementarity of the ICTs with existing services | “Many of the respondents at the workshops were generally positive about the potential of GIS as a complementary planning tool and could see its enhanced value when set against legislative and policy demands on them as purchasers and providers. They were particularly interested in the ability to present data at a number of aggregated levels simultaneously and the capability of querying the GIS to aid the planning of service provision by different age groups.”   ([Foley, 2002](#_ENREF_3)) (p. 92-93) | |
| Considering timing and family readiness for implementing the ICTs | “They document the feelings of anxiety and guilt that are frequently experienced by family carers, and recommend due sensitivity on the part of professional carers to acknowledge the complex emotions involved.  . . .  Thus, with regard to the 'Planning ahead' programme, appropriate timing according to the individual and his/her family carer is an important consideration.”   ([Hanson et al., 2000](#_ENREF_4)) (p. 718) | |
| Considering promotion strategies for respite care and the ICTs | “To better serve carers of people with dementia, this study highlights the need for promotional strategies using a wide variety of channels to provide respite service information. Greater promotion of new ‘gateway’ services, especially telephone helplines for respite information are also needed. Given the strong preference for interpersonal sources of information, the My Aged Care helpline could consider offering a named personal contact or ‘case worker’ whom carers of people with dementia can liaise with for follow up, rather than navigating their way through the Gateway system each time they call.”   ([Phillipson et al., 2019](#_ENREF_8)) (p. 6) | |

**References**

Campos-Romero, S., Herskovic, V., Fuentes, C., & Abarca, E. (2020). Perceptions on Connecting Respite Care Volunteers and Caregivers. *International Journal of Environmental Research and Public Health*, *17*(8), 2911.

Chou, L. D., Lai, N. H., Chen, Y. W., Chang, Y. J., Yang, J. Y., Huang, L. F., Chiang, W. L., Chiu, H. Y., & Shin, H. Y. (2011). Mobile social network services for families with children with developmental disabilities. *IEEE Transactions on Information Technology in Biomedicine*, *15*(4), 585.

Foley, R. (2002). Assessing the applicability of GIS in a health and social care setting: planning services for informal carers in East Sussex, England. *Social science & medicine (1982)*, *55*(1), 79-96. <http://ovidsp.ovid.com/ovidweb.cgi?T=JS&PAGE=reference&D=medc&NEWS=N&AN=12137191>

Hanson, E. J., Tetley, J., & Shewan, J. (2000). Supporting family carers using interactive multimedia. *British journal of nursing (Mark Allen Publishing)*, *9*(11), 713-719. <http://ovidsp.ovid.com/ovidweb.cgi?T=JS&PAGE=reference&D=med4&NEWS=N&AN=11235264>

McSwiggan, L. C., Marston, J., Campbell, M., Kelly, T. B., & Kroll, T. (2017). Information-sharing with respite care services for older adults: a qualitative exploration of carers' experiences. *Health & Social Care in the Community*, *25*(4), 1404-1415. <http://ovidsp.ovid.com/ovidweb.cgi?T=JS&PAGE=reference&D=med13&NEWS=N&AN=28294463>

Min, A., Currin, F., Razo, G., Connelly, K., & Shih, P. C. (2020). *Can I Take a Break? Facilitating In-Home Respite Care for Family Caregivers of Older Adults* American Medical Informatics Association Annual Symposium Proceedings (AMIA ’20), Virtual Symposium. <https://patshih.luddy.indiana.edu/>

Neef, N. A., Trachtenberg, S., Loeb, J., & Sterner, K. (1991). Video-based training of respite care providers: an interactional analysis of presentation format. *Journal of applied behavior analysis*, *24*(3), 473-486. <http://ovidsp.ovid.com/ovidweb.cgi?T=JS&PAGE=reference&D=med3&NEWS=N&AN=1836458> <https://www.ncbi.nlm.nih.gov/pmc/articles/PMC1279598/pdf/jaba00021-0077.pdf>

Phillipson, L., Johnson, K., Cridland, E., Hall, D., Neville, C., Fielding, E., & Hasan, H. (2019). Survey of knowledge of respite services. Knowledge, help-seeking and efficacy to find respite services: an exploratory study in help-seeking carers of people with dementia in the context of aged care reforms. *BMC Geriatrics*, *19*(1), 2. <http://ovidsp.ovid.com/ovidweb.cgi?T=JS&PAGE=reference&D=prem&NEWS=N&AN=30616592>

<https://www.ncbi.nlm.nih.gov/pmc/articles/PMC6322308/pdf/12877_2018_Article_1009.pdf>

Ryan, T., Noble, R., Thorpe, P., & Nolan, M. (2008). Out and about: a valued community respite service. *Journal of Dementia Care*, *16*(2), 34-35. <https://proxy.library.mcgill.ca/login?url=http://search.ebscohost.com/login.aspx?direct=true&db=rzh&AN=105739823&site=ehost-live>
